# Decentralizing video copyright protection: a novel blockchain-enabled framework with performance evaluation

**DOI:** 10.3389/frai.2025.1655709

**Published:** 2025-08-18

**Authors:** Sri Lakshmi Madapati, Nihar Ranjan Pradhan

**Affiliations:** ^1^School of Computer Science and Engineering (SCOPE), VIT-AP University, Amaravati, Andhra Pradesh, India; ^2^School of Computer Science and Engineering, VIT-AP University, Amaravati, Andhra Pradesh, India

**Keywords:** perceptual hashing, InterPlanetary File System, similarity analysis, video copyright protection, blockchain

## Abstract

**Introduction:**

Digital content, including images and videos, is increasingly ruling the online world, and so multimedia services form a part of this modern life. However, the digital resources face significant problems, especially regarding copyright infringement. In such an instance, any modification without authority infringes intellectual property rights.

**Methods:**

Based on Inter Planetary File System (IPFS) and blockchain technology, a decentralized and distributed framework has been proposed in this study for dealing with insecurity over digital assets and openness of multimedia resources. In this respect, secure, transparent, and immutable transactions in regard to the transfer and ownership of creative works have been facilitated by the use of such a framework.

**Results:**

This paper proposes novel decentralized and Blockchain enabled framework to address the problem of video copyright protection by employing solidity based smart contract in a Ethereum network, that allows the content creators to register their videos. The designed smart contract performs copyright checks and release copyright disputes by generating and comparing perceptual hash’s (Phash) for original video and modified video.

**Discussion:**

Phash techniques play a crucial role in multimedia content analysis, particularly in verifying the integrity and similarity of the video data under various transformations. Additionally, the framework generates Inter Planetary File System (IPFS) main values that signifies the ownership of the video content. Then it compars the phash values, IPFS and similarly score in public Blockchain environment i.e. Ethereum. The framework performance was measured by simulating the contracts of the Application Binary Interface (ABI), JSON file in the Hyperledger Caliper environment. This result shows the performance in the form of video registration, the measured latency was 5.02 seconds with a throughput of 409.87 seconds. For video verification the latency was 4.57 seconds with a throughput of 484.23 seconds.

## 1 Introduction

Today, the Internet is the main source of information for everyone. If we need anything, we simply get it by browsing the data, such as text, audio, video, or images, and everything is available. However, we are missing the originality of the data. In the 1920s, television and video content started gaining popularity. At that time, rebroadcasting programs and films became common, which later raised concerns about copyright protection in 1984. The “Betamax case” addressed the legality of recording content for private use (Rani et al., 2024).

The historical development from 1920 to the present covers the period up to 2025. Broadcasting television and video content has become extremely popular, but the unlawful rebroadcasting of movies and programs remains a significant challenge (Kumar et al., 2021). VSRs were invented between the 1950s and 1970s, enabling the copying of movies and programs. This eventually led to the” Betamax case,” which confronted the legality of recording and broadcasting content. From the 1990s to the 2000s, DVDs and the Internet were used for peer-to-peer file sharing through platforms such as Napster, LimeWire, and BitTorrent (Wu et al., 2022). From 2010 to the present, streaming platforms have grown in popularity. However, unauthorized uploading and distribution of videos have become a major concern. Video uploaders are identified based on their ID’s, but the the copyright regulations remains imperfect. Unauthorized screen recordings and live streaming pose significant challenges. Using such content, individuals often alter the original content due to the availability of video editing software. Blockchain is a decentralized digital ledger that records transactions across several computers while ensuring security, transparency, and immutability (Ma et al., 2018). Each transaction is organized into a block, which is linked to the preceding block by cryptographic hashes, resulting in a chronological “chain” of blocks. Based on its properties, blockchain may provide more security.

However, blockchain provides a safe, transparent, and efficient solution for managing video copyright, empowering creators, combating piracy, and assuring fair pay by using both public and private blockchains. A public blockchain is a decentralized and permissionless network that anyone can access; it is completely open and transparent. It offers global accessibility, allowing anyone with an internet connection to use it (Lee et al., 2021; Garba et al., 2021). The main problems with public blockchains are scalability issues, energy consumption, and privacy concerns. A controlled environment is provided by private blockchains. A private blockchain also faces some issues such as centralization, scalability issues, security risks, high costs, governance difficulties, compliance requirements, privacy concerns, trust issues, and limited innovation. These can be solved through efficient protocols, strong security, cost-saving solutions, privacy tools, and other measures (Guo et al., 2020). A public blockchain is an open, permissionless network that anyone can join to read, write, and validate transactions. It is completely decentralized, with examples including Ethereum and Bitcoin. Blockchain technology provides trust and immutability. In contrast, a private blockchain is a decentralized, permissioned network that is controlled by a single organization or a group of organizations. Only approved users can view or authenticate transactions, providing privacy control. Due to this, it is less decentralized than a public blockchain (Yang et al., 2020; Wang et al., 2023).

Therefore, a perceptual hash (pHash) function is a neat solution to this problem. A perceptual hash function has the characteristic that the hash of an original input is associated with the hash of a slightly altered input. As a result, perceptual hashing can be used to detect manipulated picture and video frames without substantially changing how they look. The distributed hash table (DHT) technique is a well-known feature of the peer-to-peer file sharing network, the InterPlanetary File System (IPFS). A unique 46-byte hash is assigned to every file, including images and videos, to maximize storage and retrieval (Hasan and Salah, 2019). The IPFS hash value is changed when the content creators modifies the image or the video frame in the distributed hash table. Features such as immutability, integrity, dependability, and transaction availability are provided by blockchain technology (Dave et al., 2022). Each block that makes up a blockchain contains a number of transactions (Gürfidan and Ersoy, 2021). Our method involves storing the perceptual hash of every picture or movie shared on the blockchain network’s IPFS distributed file-sharing system (Li and Wang, 2016; Lee and Choi, 2022; Singh and Aggarwal, 2018; Yu et al., 2022; Gangwar et al., 2018).

We propose a solution to the problem of video copyright protection—the use of IPFS-based file sharing and blockchain technology. Blockchain-based solutions have a lot to offer in terms of preventing video copyright violations. We propose a blockchain-based distributed system to prevent copyright violations in videos by utilizing the pHash algorithm. Below is an overview of the contributions made by our study:

### 1.1 Major contributions

- We recommend a blockchain-based and IPFS-based decentralized platform to store videos as transactions, providing copyright protection, availability, immutability, and transparency through peer ledgers and consensus mechanisms.
- Our decentralized system stores videos on Ethereum and establishes immutable ownership records, while smart contracts automate copyright verification based on perceptual hashes, thereby being transparent and time-saving and eliminating intermediaries.
- The decentralized blockchain system guarantees clear, secure, and verifiable ownership records of the video in a transparent manner and automatically identifies copyright conflicts through perceptual hash comparison. It provides a transparent, blockchain-based resolution process.
- It provides a user-friendly UI for creators, users, and other stakeholders to interact with this platform. The platform offers a user-friendly interface, making copyright protection easy and scalable for both individuals and large organizations.

### 1.2 Organization

The structure of the paper is as follows: Section 2 deals with the background. Section 3 presents the details of the study. Section 4 describes the proposed approach, and Section 5 deals with the implementation details. Section 6 shows the results and performance analysis of the framework. Finally, Section 7 concludes the study and discusses future work.

## 2 Background

Hashing techniques are very effective for storing, retrieving, and managing data. There are four types of hashing techniques related to images: wavelet hashing (wHash), pHash, difference hash (dHash), and average hash (aHash).

### 2.1 pHash (perceptual hash)

This pHash uses the discrete cosine transform (DCT) to capture the low-frequency components of an image. The hash value is generated using perceptual characteristics rather than the pixel value. pHash works well with the image modifications i.e minior changes are also able to identify. pHash is focused on frequency and not on pixel intensity. Therefore, it handles brightness or contrast changes easily. It is very effective for detecting similarities between the images. In addition, it is less affected by the noise. pHash is more effective because it needs more processing power compared to aHash and dHash. Therefore, pHash is mainly used for reverse image and for detecting edited images.

pHash has the ability to capture the perceptual characteristics of video frames, making it robust to common modifications, including minor ones such as resizing and compression. It allows us to measure content similarity, which is the initial step in our framework for detecting altered copies. It also provides greater efficiency compared to other hashing techniques such as wHash, aHash, and dHash, and it is well-suited for video comparisons. Due to these reasons, we chose the pHash technique for frame conversion.

### 2.2 wHash (wavelet hashing)

wHash uses the Haar wavelet transform to convert an image into frequency components. The wHash technique is similar to the pHash technique; however, it works better with image modifications such as resizing, blurring, and noise. In addition, wHash provides better structural data, such as comparing two images with textures or patterns. It is significantly less sensitive to brightness changes compared to other hashing techniques. wHash is very effective for high-quality image retrieval, particularly in forensic applications. Therefore, the wHash technique is useful for identifying image similarities, retrieving high-quality reverse images, image forensics, and content matching.

### 2.3 dHash (difference hash)

The dHash technique uses the grayscale value. First, it converts an image to a grayscale image; then, it resizes it to 9*8 pixels and compares it with its adjacent pixels. Based on brightness, it assigns 1 or 0 to each pixel; for instance, if the left pixel is brighter than the right pixel, it assigns 1 to it, otherwise it assigns 0. In large-scale image datasets, the dHash technique is useful and faster compared to pHash. It is very good at detecting minor modifications in an image. The dHash technique works well even if images are resized because it is less sensitive to compression and scaling. When an image is cropped, the hash value changes because it is sensitive to the robustness of the image. It is very sensitive to brightness and contrast changes because this hashing technique depends directly on pixel intensity differences. It is not effective for detecting complex transformations, such as adding filters to an image or object movement. pHash is mainly useful for detecting small modifications and fast duplicate image detection only.

### 2.4 aHash (average hash)

aHash also converts an image to a grayscale image, resizes it to an 8*8 image, and calculates the average intensity. Each pixel value is compared with the average value. If the pixel value is above average, it assigns 1 to the pixel; if the pixel value is below average, then it assigns 0 to the pixel. The aHash technique is the fastest method for quick compression by producing similar hash values.

It is highly sensitive to brightness. If brightness is adjusted, the hash value also changes. In addition, it is not effective for cropped and rotated images. dHash is dependent on the pixel intensity value and is not effective when noise is added to images. aHash is only good for finding exact duplicates in an image and lightweight applications.

These techniques are used for detecting visual similarities between videos. To enable a decentralized system, we use the IPFS and Swarm. For storing and accessing data without a central authority, the IPFS and Swarm are used. These provide decentralized, distributed file storage for blockchain-based applications.

### 2.5 IPFS (InterPlanetary File System)

The IPFS is a peer-to-peer distributed file system. The main aim of the IPFS is to store and share data in a decentralized manner. It uses the CID (Content Addressing), and because of this feature, it provides unique cryptographic hashes for each file. For representing files and directories, it uses DAG. By using this, large data are split into smaller parts, and each part is connected with links. Duplication does not occur because every file has its own address. The IPFS uses the content in the distributed system. It is mainly used in the sharing of files, DApps, websites, etc.

### 2.6 Swarm

Swarm is an Ethereum-native decentralized storage system. Similar to the IPFS, Swarm also splits files into smaller parts and addresses them, and it also supports updatable feeds. In this system, the filters are updated with a stamp only (proof of payment). BZZ tokens are provided as rewards for storage nodes for motivation. This system supports mutable files and real-time updates. It is suitable for hosting entire DApps, websites, and more. However, setting up the system is more complex compared to the IPFS.

## 3 Related work

With the emergence of blockchain technology, new solutions have arisen to address challenges in digital copyright and Internet business, including video content production. Researchers have argued that conventional means of IP management, including digital watermarking, encryption, and DRM, fall short in providing adequate security and authentication for digital assets. Such centralized systems may be manipulated, pirated, and broadcast without the consent of the creators of such systems.

Blockchain, as designed, provides a shared, decentralized, transparent, and immutable system for operational record-keeping that addresses many of these challenges. Using the concept of blockchain, video content could be registered securely, and the ownership of content could be virtually defensible as it is stored in a decentralized manner. This, in a way, allows creators to assert their ownership of the content, access the path of the material, and prevent piracy. Moreover, the integration of perceptual hashing with blockchain has benefits for security, as video fingerprints can be easily distinguished and stored on the blockchain, decreasing the occurrence of piracy.

The current literature review in Table 1 shows that despite the many opportunities blockchain offers in the area of copyright protection, there are many barriers to its effective implementation. Among these promising directions, challenges such as the workability of blockchain for big data, the problem of integration of various blockchain platforms, and the legal regulation of blockchain in the field of intellectual property management stand out. The majority of established blockchain-based copyright protection systems are still under development and remain unproven in real-world conditions. Several projects are currently exploring how various forms of blockchain can be used for content protection. Some projects focus on improving the video watermarking method, while others combine blockchain with smart contracts when it comes to the automation of licensing and copyright infringement. However, to date, the application of blockchain technology in the management of copyrights remains limited due to technical restrictions such as the rate of transactions, energy consumption, and legal or regulatory constraints. Thus, blockchain-based systems present significant potential for the protection and management of copyrights. However, for a more comprehensive solution, it is crucial to focus on further improving the implementation of blockchain solutions in the management of copyrights. More studies are emerging on how this new technology can complement present copyright frameworks, with the goal of designing a stronger and more effective mechanism to safeguard copyright materials, especially in modern societies where content sharing is rapidly increasing.

**Table 1:** Blockchain and digital content applications.

References	Year	Application	B	S	T	SC	CP
1	2024	Blockchain and non-fungible tokens for educational resources	✓	*×*	*×*	✓	✓
2	2021	pHash and the IPFS for images and videos	✓	✓	✓	*×*	✓
3	2022	NSCT-SVD-based zero watermarking for video copyright	✓	✓	*×*	*×*	✓
4	2018	DRM chain for digital content	✓	*×*	*×*	*×*	*×*
5	2021	SPDC for digital content	✓	*×*	*×*	*×*	✓
6	2019	Smart contract for digital multimedia resources	✓	✓	*×*	*×*	✓
7	2019	Ethereum smart contract for the provenance of digital media	✓	✓	*×*	*×*	✓
8	2021	Blockchain for smart home environments	✓	*×*	*×*	*×*	*×*
9	2021	Blockchain for multimedia content	✓	*×*	*×*	✓	*×*
10	2021	Blockchain for music wallets	✓	*×*	*×*	*×*	*×*
Proposed		Blockchain for video copyright protection	✓	✓	✓	✓	✓

*B, blockchain; *S, security; *T, trust; *SC, scalability; *CP, copyright protection.

## 4 Proposed system model for video copyright protection

### 4.1 System architecture

The development of digital media has also posed challenges in the protection of video content from copyright infringement. Traditional systems of copyright management are usually centralized, easy to tamper, and not accessible to most creators. Blockchain is an innovative approach to tackle the problem of counterfeiting and provides a secure platform for the registration of video ownership and the verification of copyright in a decentralized and secure manner. This article presents a proposed framework that implements perceptual hashing of video content and leverages smart contracts on the blockchain to manage and safeguard the registration, verification, and protection of the content. The Ethereum smart contract stores videos by recording their pHash, IPFS hash, and owner address; it checks new videos by comparing pHash values to identify copyright infringement and stores disputes on the blockchain, making ownership secure, transparent, and tamperproof. For the back end, the platform uses Python Flask, and for application program interface (API) testing, the platform uses Postman. The platform interacts with the Ethereum blockchain to securely store and manage ownership data. This system gives power to content creators to safeguard their intellectual property more efficiently and transparently.

The proposed system includes two smart contracts: the Contract Video Registry, used for registering videos, and the ABI of Contract, used for authenticating videos. A mapping function is used to establish a key value data structure, allowing for effective storage and retrieval of values. The function supports conversions, such as from byte32 to video, and computations within the Contract Video Registry. It also plays an important role in the decentralized video-sharing application by simplifying data access and processing.

The video is converted into an *n* number of frames of equal size, and a perceptual hash (pHash) value is computed for each frame to represent its visual features. pHash captures the perceptual content and identifies visually similar frames. It can also detect frames that have undergone transformations such as resizing or compression. We also utilize this pHash for similarity matching by calculating the Hamming distance between the hashes, making pHash highly effective for the detection of duplicate video content.

**Figure 1:**
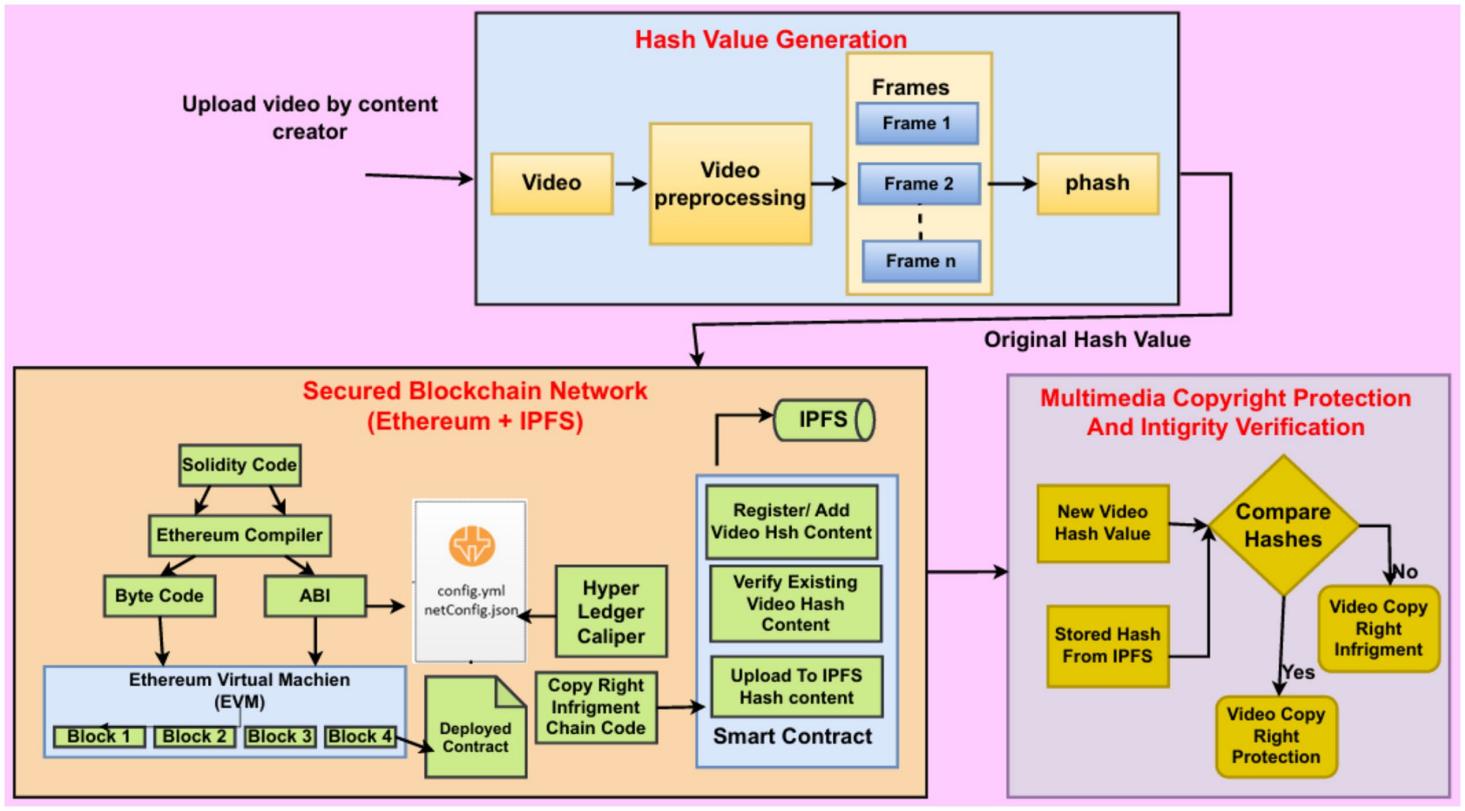
Proposed system model for video copyright protection.

The main aim of the proposed system model is to ensure secure, tamper-proof, and decentralized video copyright protection using perceptual hashing, the IPFS, and blockchain technology. The architecture mainly contains three functional modules: Hash Value Generation, Secure Blockchain Network, and Multimedia Copyright Protection, as shown in Figure 1.

1. **Hash Value Generation**: When a video is uploaded by a user initially, a unique digital fingerprint of the video gets generated. This video then undergoes preprocessing, where it is divided into frames based on a time sequence for processing. PHash is generated for each and every frame. A frame-by-frame comparison is conducted using pHash. These hash values represent the visual essence of each frame. Instead of storing and comparing entire frames, the system calculates and stores their hash values only (64bit per frame). During the verification step, the uploaded video goes through the same pre-processing and hashing process. These hashes are then compared with the stored hashes using the Hamming distance to measure similarity. This makes it possible to effectively detect similarities, even small modifications such as resizing and compression. This approach achieves a balance between robustness and resource efficiency, making it scalable for video copyright protection. The final hash values represent the original hash value of a particular video, and this original hash value is used for verification and protection against copyright infringement.
2. **Secured Blockchain Network:** Here, the blockchain network is created using Ethereum and IPFS technologies. This network enables secure registration and verification of video hashes through blockchain smart contracts and decentralized storage.

- **Smart Contract Deployment:** A smart contract in Solidity is written and compiled. After deploying this code on the Ethereum Virtual Machine (EVM), both the bytecode and application binary interface (ABI) are generated for decentralized execution.
- **Hyperledger:** Hyperledger handles the actual video copyright ledger. The system employs a configuration file (e.g., netConfig.json) to establish and keep the blockchain network. The recorded video hashes are safely stored on this ledger.
- **IPFS:** Metadata are stored on the IPFS in a .bib file to provide decentralized and distributed storage. The pHash and IPFS hash are recorded and stored on the blockchain to create tamper-proof records.

1. **Multimedia Copyright Protection and Integrity Verification:** Multimedia provides continuous surveillance and enforcement of copyright protection.

- **Comparison Process:** Whenever a new video is uploaded, its perceptual hash value is calculated and compared with existing pHash values on the blockchain network.
- **Verification and Decision:** Based on the similarity threshold (i.e > =50%), the system determines whether a video is duplicated or unauthorized. If the video matches an existing one in the network, it is marked as copyrighted and flagged as a copyright infringement. If the video is new, it is eligible to be added to the blockchain network along with its IPFS records.

The proposed framework ensures the security of videos and operates using Algorithms 1–3.

- Algorithm 1: This algorithm is used for registering videos on the Ethereum network. For storing videos in the metadata, we use two parameters they are IPFS hash and address of the uploader. Unique ID is created for every video after that it checks the hash value to know that the video is already registered video or the new one.

- Algorithm 2: This algorithm verifies whether a video already exists or is new using jsonify from Flask. The backend program of our proposed framework is shown in Algorithm 2. It is a Flask-based backend to interact with the Ethereum network. In addition, the IPFS is used for providing security by supporting video registration and verification. The video is registered on the blockchain using a smart contract. Using the IPFS and pHash values, the system verifies whether a video has already been registered. It provides three functions: registering, verifying, and comparing. All of these are performed based on user requirements.

- Algorithm 3: This algorithm outlines the steps for registering, verifying, and comparing videos. The Postman API facilitates communication among all participants and ensures transparency and security by supporting the creation of video authenticity records. The main purpose of this algorithm is to compare two videos using their IPFS and pHash values. In addition, it extracts audio features from both videos using movie.py to compare their similarity. A threshold value is used to find the difference between frames and audio. By comparing these three parameters, the algorithm identifies whether the input video is identical to an existing one or has been edited.

**ALGORITHM 1:**
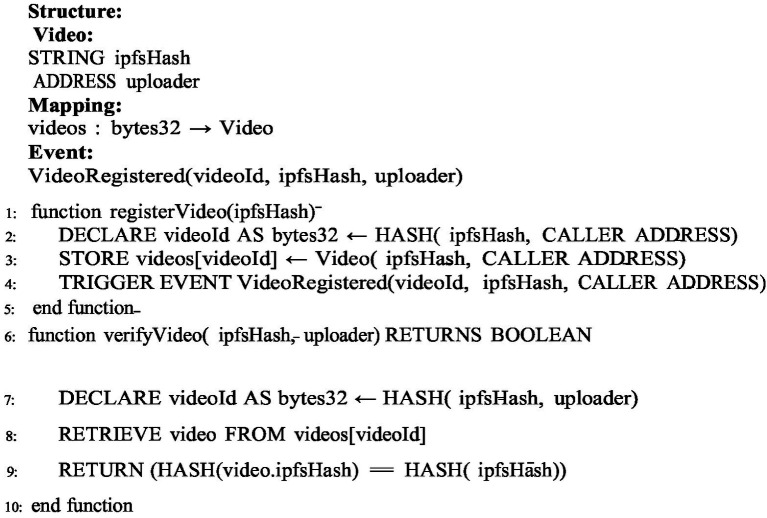
Video registry.

**ALGORITHM 2:**
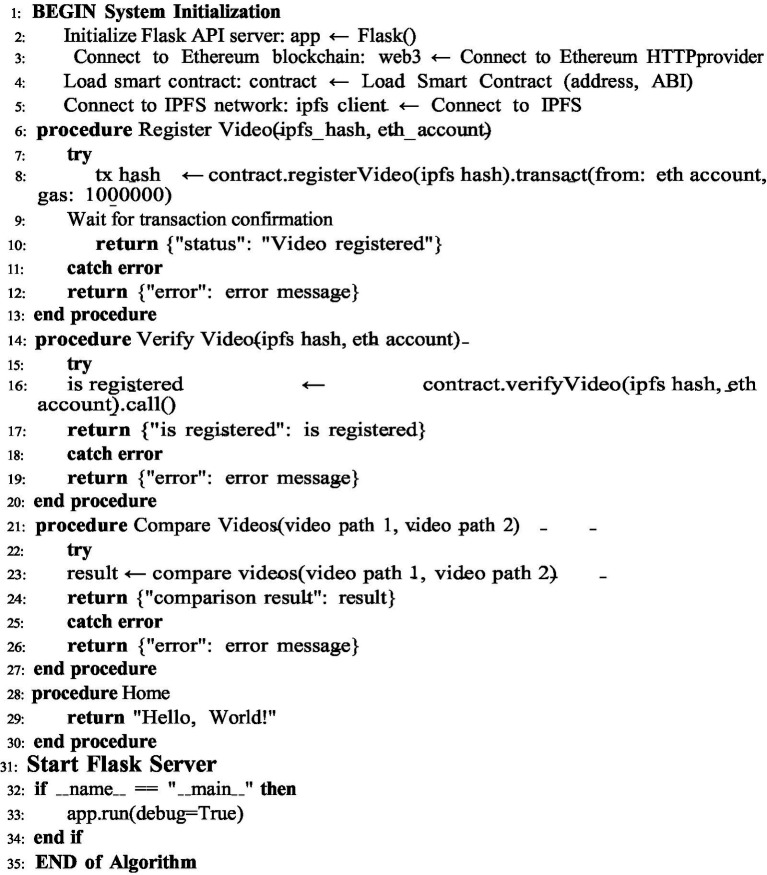
Perceptual hash-based video content identification.

**ALGORITHM 3:**
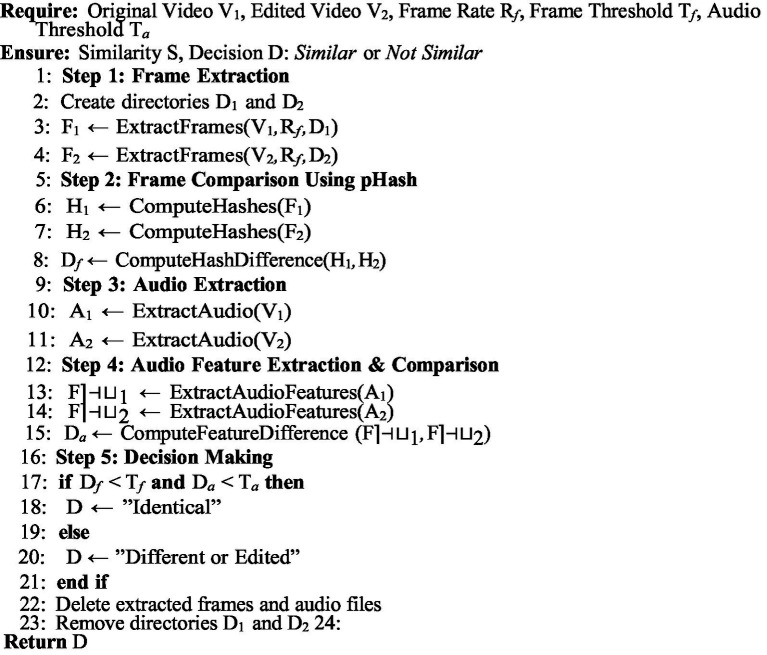
Video similarity comparison.

**Figure 2:**
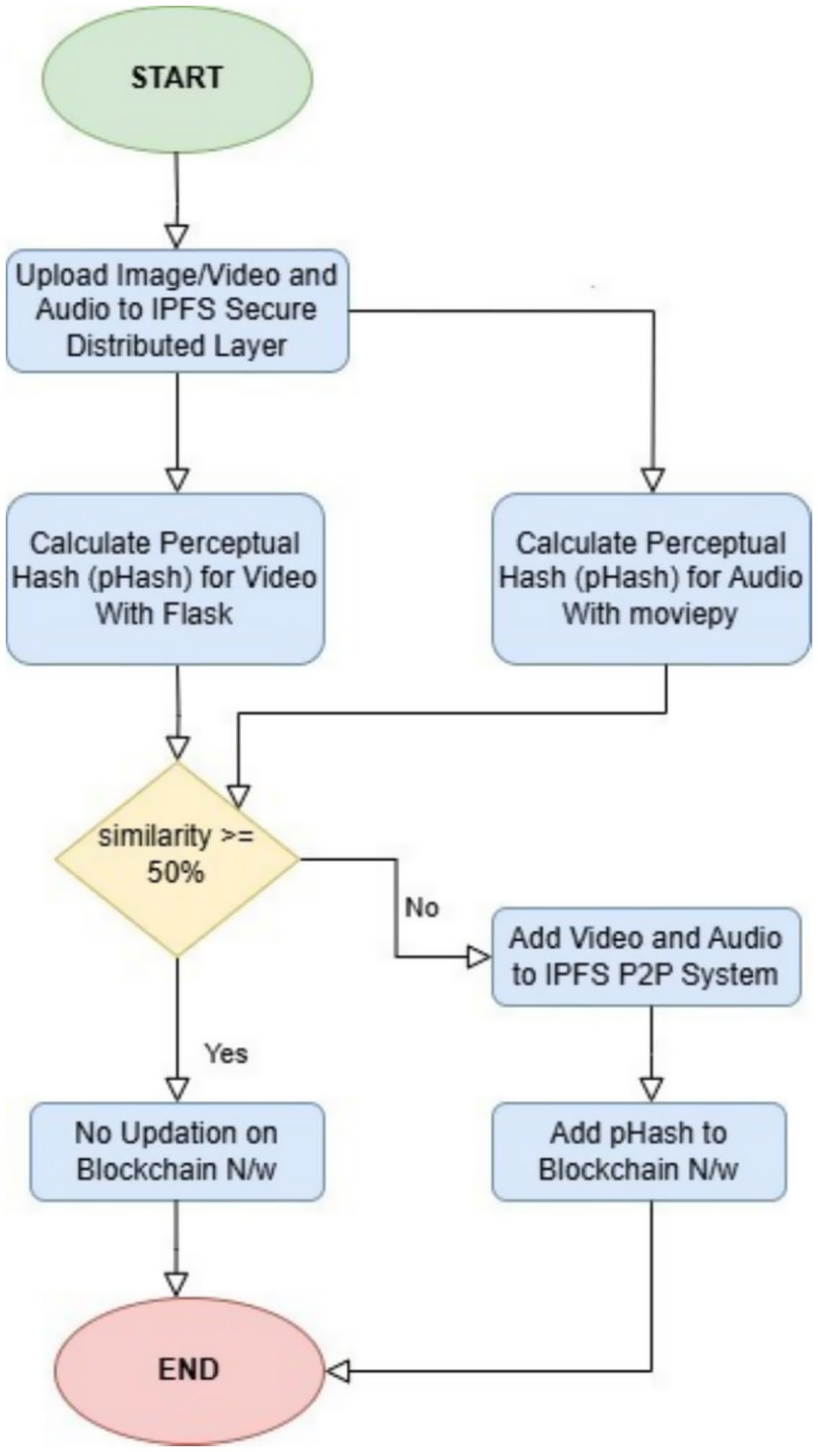
System block diagram.

To better understand the process, we designed a flowchart that visually explains the execution flow. Figure 2 presents a block diagram illustrating the workflow of our system (Kumar et al., 2021). When a new video is added to the decentralized network, its pHash value and IPFS hash are computed. These values are then compared with those of existing videos in the network. If the similarity exceeds 50%, the video is not added to the blockchain. However, if the similarity is below this threshold, the video is successfully added to the blockchain network.

## 5 Implementation

The system utilizes the Ethereum blockchain to securely register and verify video ownership. Table 2 lists the software used for the implementation. Video creators upload their content, and Postman is used to interact with backend APIs, which generate a unique perceptual hash of the video. This hash, a digital fingerprint, is then stored on the blockchain to provide an immutable record of ownership. Key frames are extracted from the video for efficient hashing, ensuring minimal computational overhead. When ownership verification is required, perceptual hashes are compared through smart contracts, automating the process of detecting unauthorized use or ownership disputes. If a mismatch occurs, the system flags the content for further review, ensuring transparency and reducing reliance on third-party intervention. The backend, built using Python and Flask, ensures smooth communication with the blockchain, while Postman is used for testing and interaction with APIs. MetaMask is integrated to manage user authentication securely, allowing only authorized users to perform actions such as video registration or claim ownership. Moreover, the Ethereum blockchain ensures that ownership data are decentralized, and hence, secure, and transparent, thereby instilling confidence in users regarding the authenticity of recorded ownership. The approach combines the security of blockchain with the efficiency of perceptual hashing to create a robust video copyright management system that provides enhanced security and transparency in handling digital content for both creators and consumers alike.

**Table 2:** Software requirements and specifications for the proposed video copyright protection system.

Tool/technology	Version
Solidity	0.8.9
VS code	1.68
Truffle suite	5.4.6
Ganache	v6.12.1
MetaMask	10.1.0
Python (flask)	4.3.4
Postman API	9.1.1
Solidity extensions for VS Code	0.0.88
Java Script	9.1.1

### 5.1 Testbed setup

The system integrates a Python Flask-based backend with the Ethereum blockchain to provide decentralized video copyright protection. The backend handles user authentication, perceptual hash generation, and communication with the blockchain. Postman is used for testing APIs that manage video registration and ownership verification processes. The Ethereum blockchain ensures secure, immutable storage of perceptual hashes, while smart contracts automate ownership verification and dispute resolution. MetaMask manages user authentication, enabling secure blockchain interactions. This combination of technologies provides a scalable and reliable framework for video copyright management. The software components of the project are designed to ensure secure, efficient, and decentralized management of video copyrights. The backend is developed using Python Flask, which handles API requests for video registration, perceptual hash generation, and blockchain interactions. The APIs are tested and interacted with using Postman, and this makes the communication between the user and the system very easy. The blockchain infrastructure is built on Ethereum, utilizing its robust decentralized framework for storing perceptual hashes and deploying smart contracts written in Solidity. These smart contracts automate processes such as ownership verification and dispute resolution. MetaMask is integrated to manage user authentication and enable secure blockchain transactions. This combination of software ensures a reliable, scalable, and transparent solution for protecting video copyrights.

## 6 Result analysis and discussion

**Figure 3:**
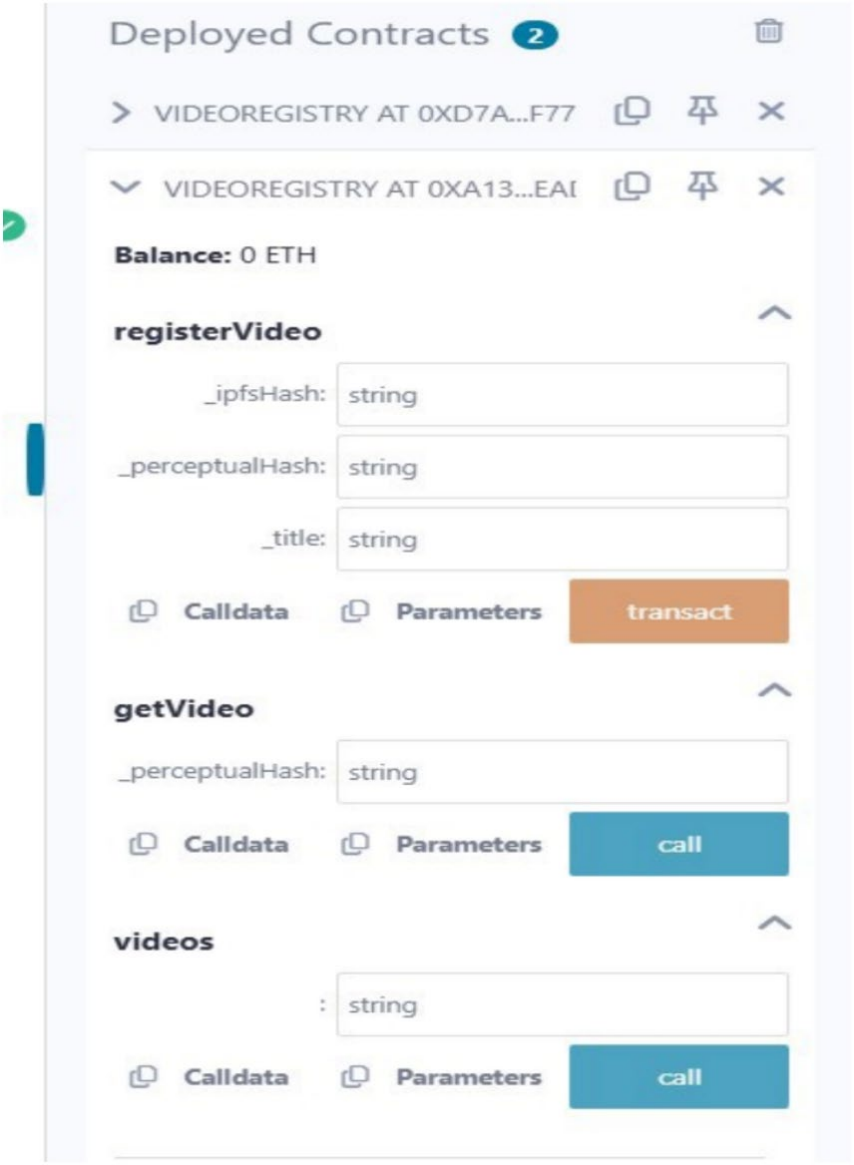
Output of the deployed smart contract on the Ethereum blockchain network.

**Figure 4:**
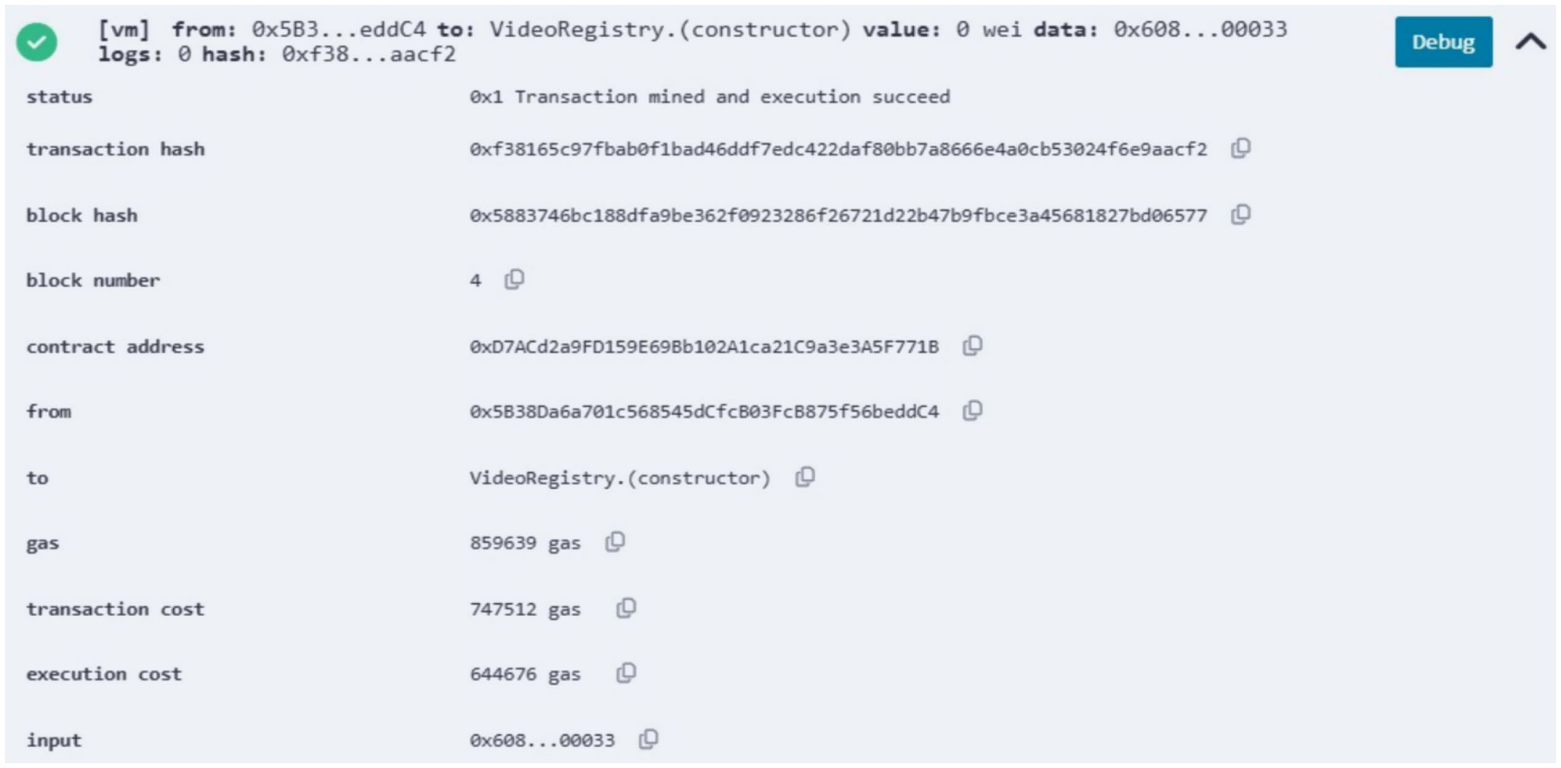
Status of the deployed smart contract.

**Figure 5:**
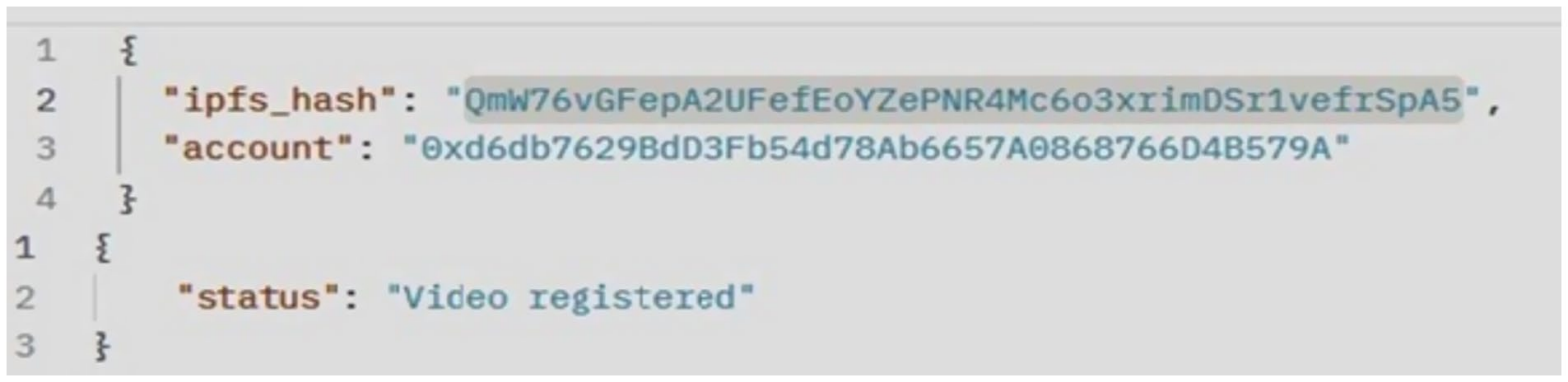
Video content registration by client (Postman API).

**Figure 6:**
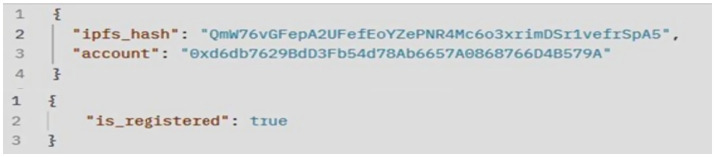
Video content verification.

**Figure 7:**
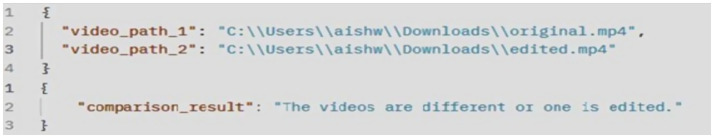
Video content comparison.

In this section, we analyze and discuss the results with respect to our proposed and implemented framework. Figure 3 depicts the result when the proposed smart contract was deployed on the Ethereum blockchain network. It shows smart contract functions such as registeringVideos, getVideo, and videos as inputs to the blockchain network. The orange-colored button indicates functions that write or update state values in the blockchain network. Similarly, the blue-colored button represents functions that read values from the blockchain network. After successful compilation with the Solidity compiler and deployment of the bytecode.

of the smart contract to the blockchain network, we obtained the transaction status, hash, block hash, block number, contract address, owner address, gas values, and bytecode, as shown in Figure 4. Client communication with the video copyright framework is facilitated through the Postman API. Figure 5 illustrates the Postman call made when a video content is registered by a content owner. As a result, it returns the InterPlanetary File System (IPFS) value, the owner’s externally owned account (EOA) address, and the status of the video registration. Figure 6 verifies whether the video content is from the original owner’s address or not. As a result, it shows the IPFS hash value, the owner’s EOA address, and whether the video is registered or not. To compare the copyright of a video, we conducted a simulation using an original video and a modified video. Figure 7 shows the output of the video content comparison, which took the path of the original and modified videos and compared their frames using their pHash values. As a result we get the video as original video or copyrighted video.

**Figure 8:**
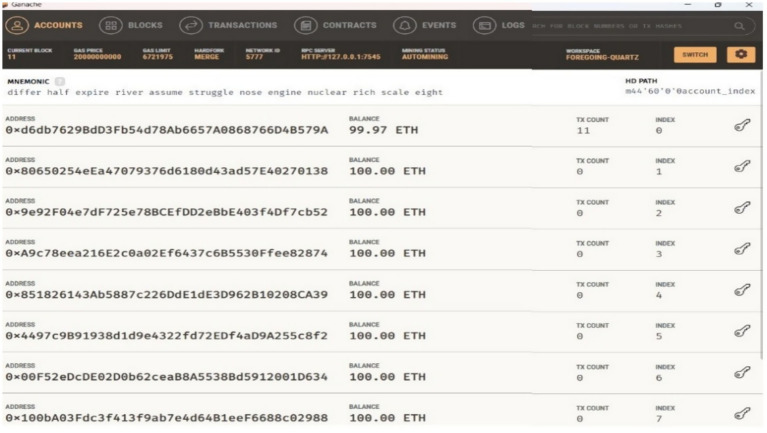
Ethereum externally owned account (EOA).

**Figure 9:**
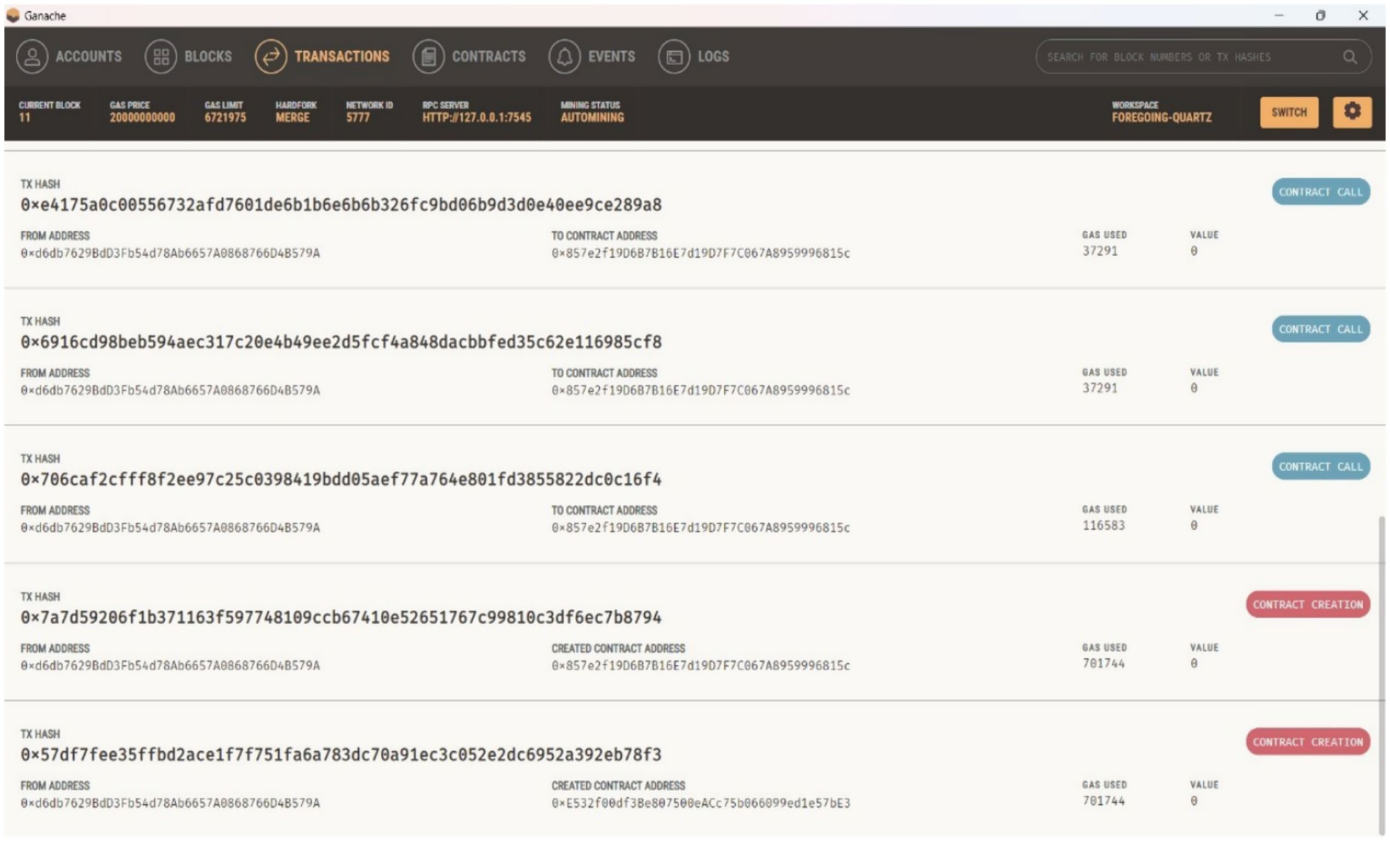
Transactions generated by the proposed framework.

**Figure 10:**
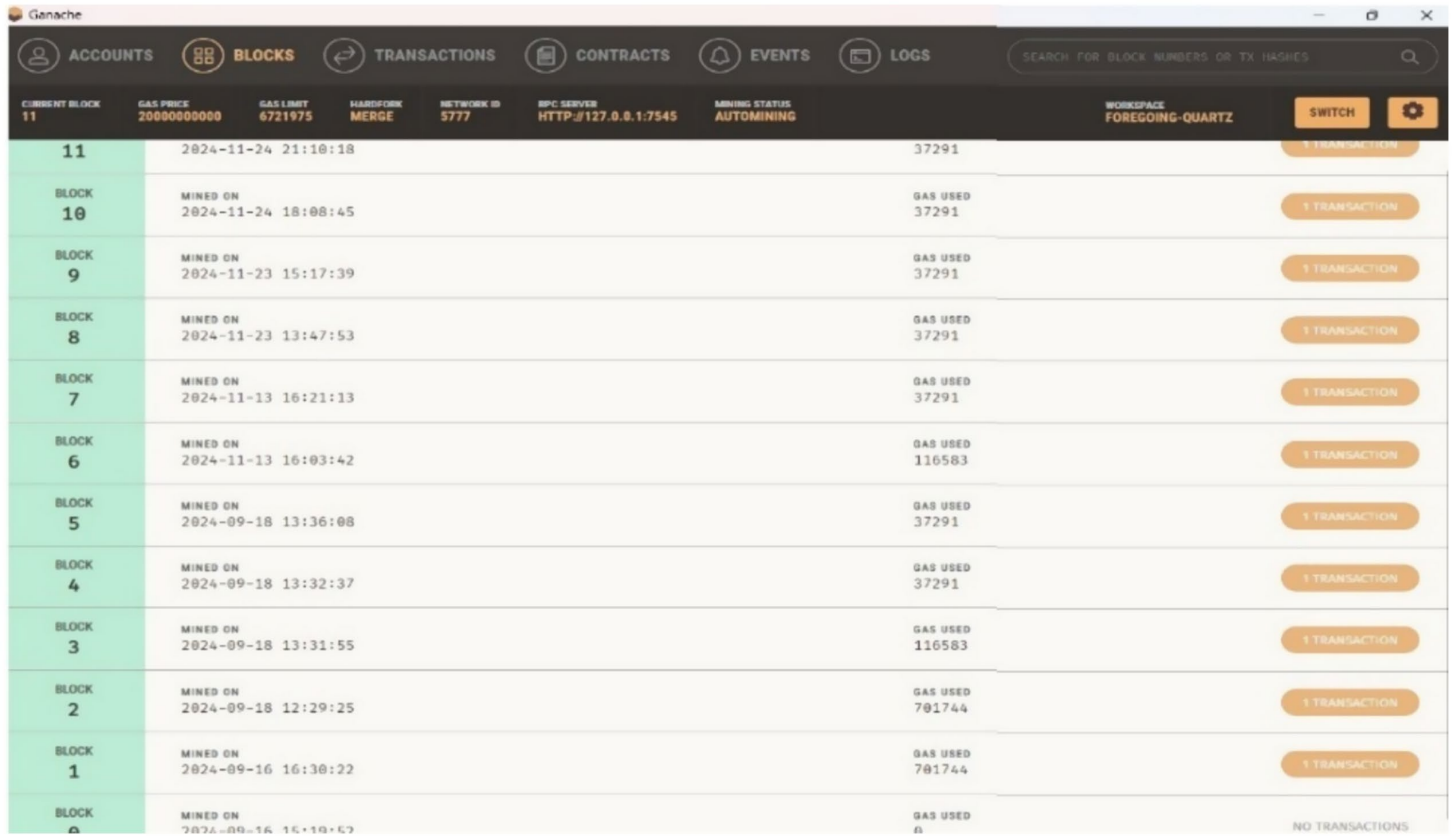
Blocks generated by the proposed framework.

Ganache is a private network used on the Ethereum platform for testing smart contracts locally. Figures 8–10 show the Ganache accounts and transactions within the Ethereum network. Figure 8 represents the EOA address, Ether account balance, transaction details, and index value. Figure 9 shows the transaction hash value, the sender’s address, the contact address, and the amount of gas used for the deployed contract. Figure 10 shows the number of blocks, a timestamp value with date and time, and the amount of gas used for automatically initiated transactions.

To analyze the framework’s performance at increased transactions and to measure its efficiency, tests were conducted on various important functionalities of the model using an EVM node. Operations such as video registration and verification were thoroughly tested at various transaction rates, and the results were noted for future reference. The same simulation was run five more times to find variations, and the average results were taken into consideration. The results are shown in Tables 3, 4. All values are rounded to two decimal places. For performance testing using Hyperledger Caliper, we conducted 5,000 transactions, with each transaction consisting of three operations: RegisterVideo, GetVideo, and CompareVideo. Each operation was tested at transaction rates of 1,000, 2,500, and 4,000 transactions per second.

**Table 3:** Framework performance based on transactions and types.

Function	Txns	Succ	TPS	Latency (Avg.)	Throughput (Avg.)
RegisterVideo	5,000	5,000	1,000, 2,500, 4,000	4.98, 5.06, 5.27	410.8, 409.6, 409.2
GetVideo	5,000	5,000	1,000, 2,500, 4,001	4.07, 4.38, 4.75	502.1, 484.6, 496.3
CompareVideo	5,000	5,000	1,000, 2,500, 4,002	4.77, 4.76, 5.03	480.8, 454.3, 486.3

**Table 4:** Performance after completion of all rounds.

Function	TPS	CPU % Max	CPU % Avg.	Max memory in MB	Avg. memory in MB	Traffic in MB	Traffic out MB
RegisterVideo	1,000	81.16	54.05	956	775	20.8	30.5
	2,500	76.30	44.67	761	709	20.8	30.5
	4,000	74.15	46.18	911	714	20.8	30.5
GetVideo	1,000	78.11	43.12	1,020	749	12.1	29.4
	2,500	80.83	44.56	952	684	12.1	29.4
	4,000	80.33	43.67	934	712	12.1	29.4
CompareVideo	1,000	74.06	42.03	955	740	12	28.5
	2,500	79.70	38.35	888	672	12	28.7
	4,000	80.49	35.92	911	698	12	28.9

**Figure 11:**
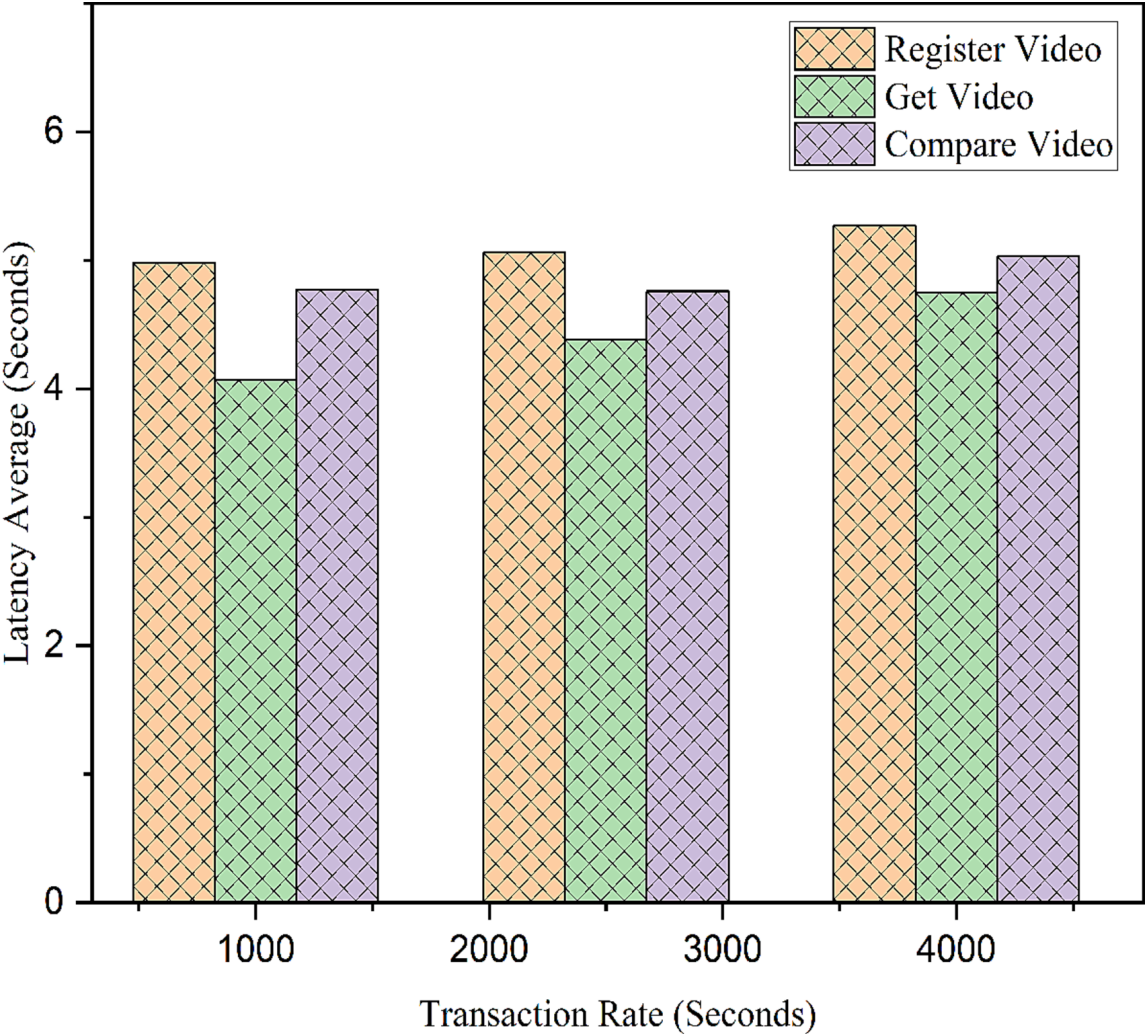
Variable transaction rate latency.

These functions exhibited consistent latency between 4.07 and 5.27 s at different transaction send rates (1,000, 2,500, and 4000TPS) for the 5,000 transactions submitted to an EVM node. Latency represents the interval between a transaction’s start and network confirmation. Figure 11 depicts that the RegisterVideo function takes more latency compared to the other subfunctions such as Get Video and Compare Video as RegisterVideo deals with hash values and IPFS values for video ownership. GetVideo is a kind of read transaction. Therefore, it has the lowest latency, that is, 4.4 s compared to the other functions and subfunctions. The Compare Video function compares the original video with the edited video/copyright-violated videos by analyzing perceptual hash and IPFS values. Therefore, Compare Video has a latency of approximately 4.85 s.

**Figure 12:**
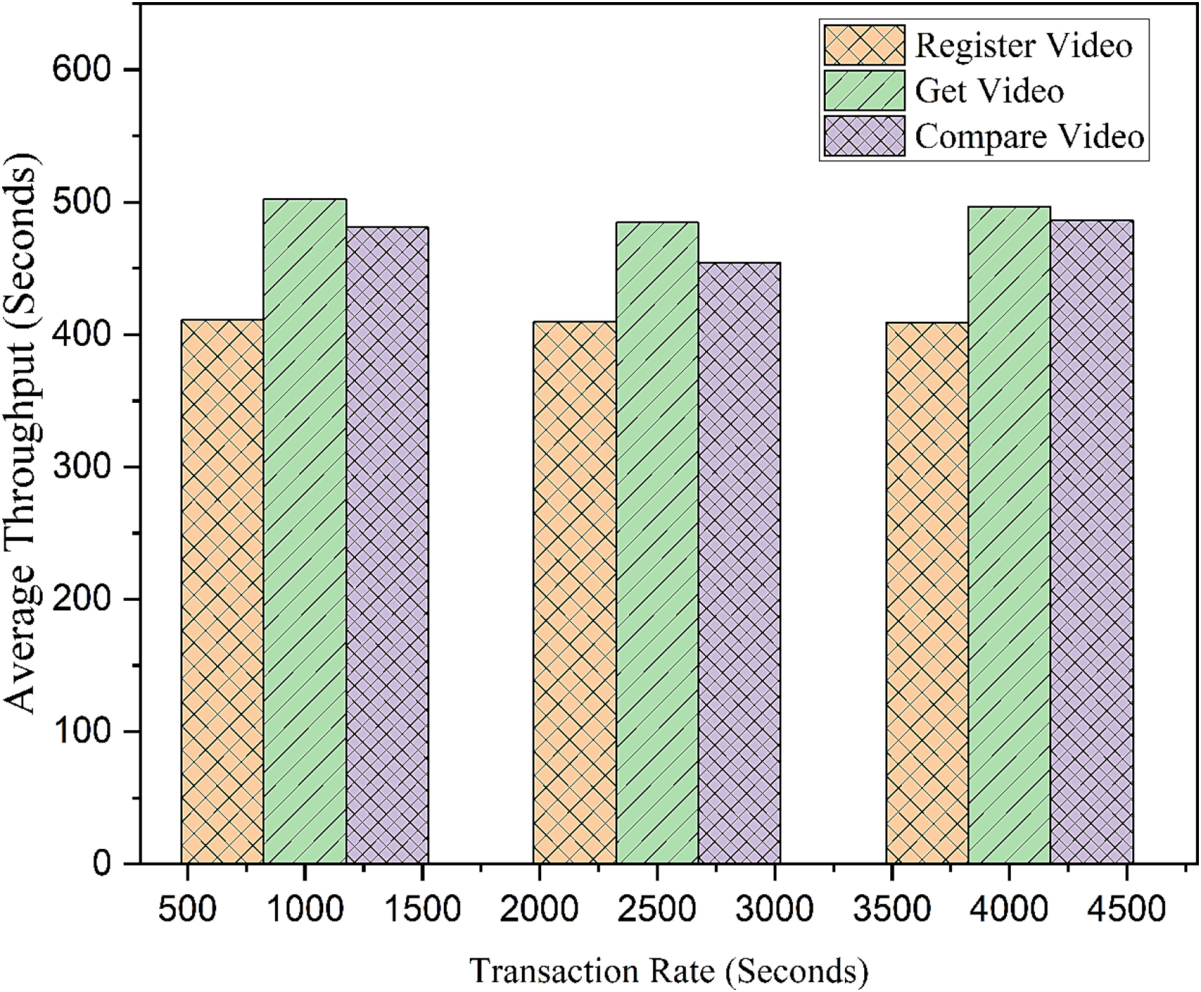
Transaction rate variation in throughput.

Furthermore, the number of transactions that are handled by a network per unit time is throughput, and the throughput analysis also demonstrated consistent behavior, with 409 to 502 transactions per second for each function. GetVideo had the highest throughput, while RegisterVideo had the lowest at the same transaction end rates. Figure 12 reveals that the read transaction, i.e., GetVideo, achieves higher throughput compared to the other functions because this function verifies the video using its IPFS hash value and the address of the uploader. Registe Video had the minimum throughput of 409.87 s due to its functionality. The Compare Video function compares the original video with the edited video by analyzing perceptual hash and IPFS hash values. Therefore, Compare Video takes 477.23 s more. The throughput graph in Figure 12 reveals that the framework performs better under higher loads.

**Figure 13:**
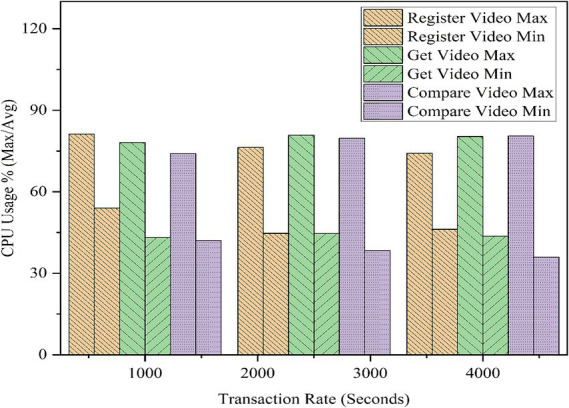
CPU usage.

**Figure 14:**
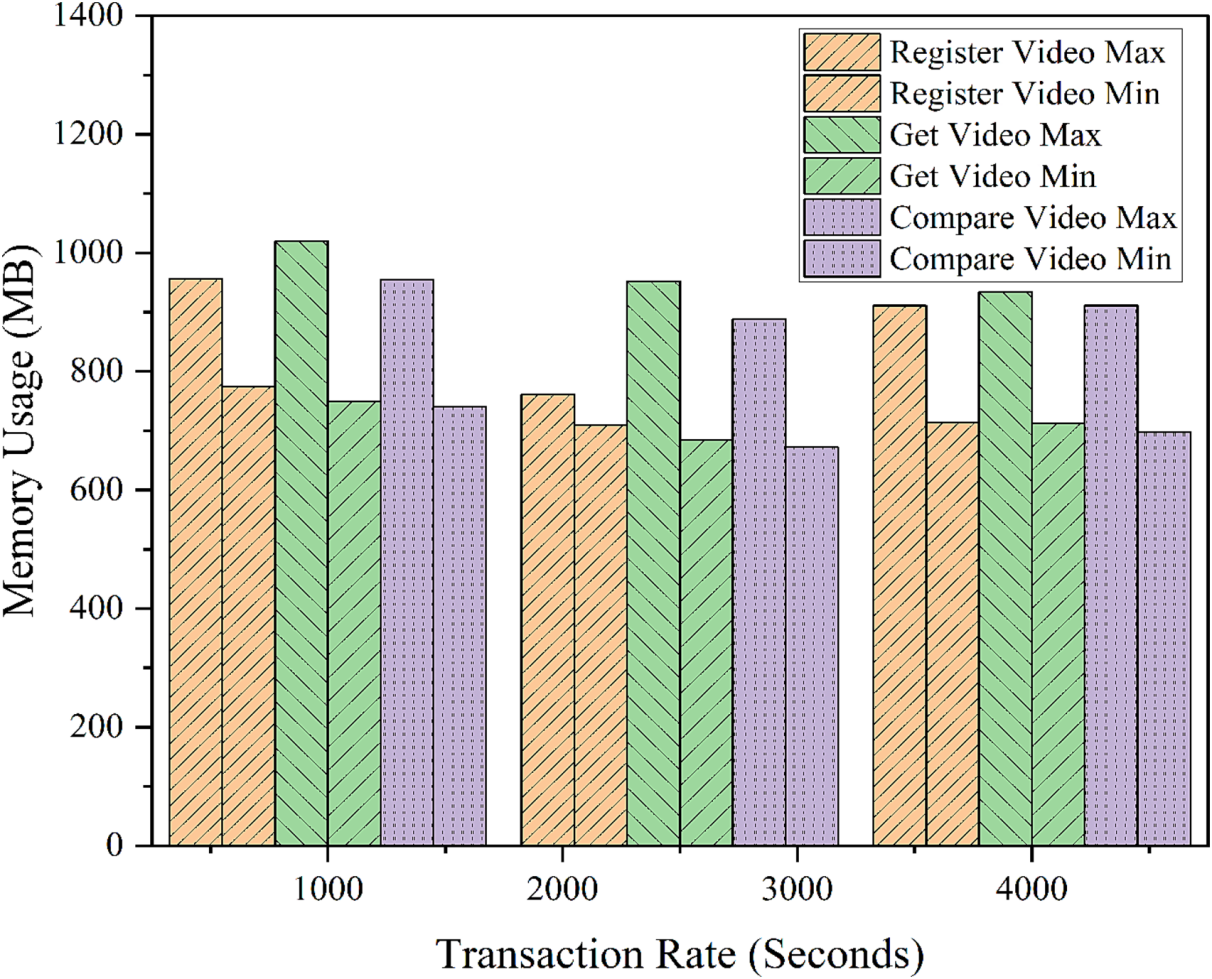
Memory usage.

**Figure 15:**
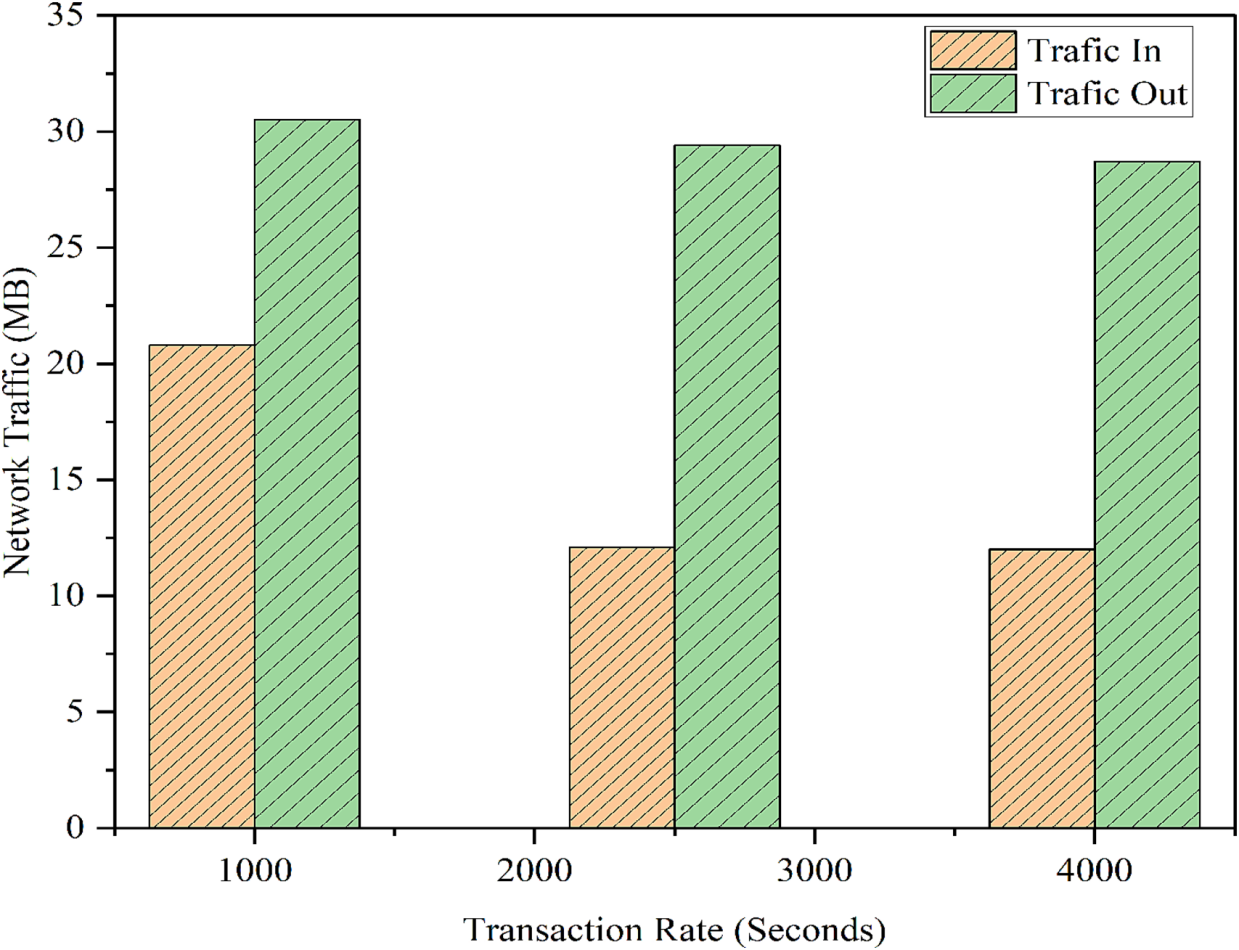
Network traffic.

In addition, CPU, memory, and traffic resource usage data were computed and examined. The parameters were examined during the same 5,000 transactions, under transaction input rates of 1,000, 2,500, and 4,000 TPS. The highest CPU usage of the framework was 81.16%, and the average CPU usage was 35.92%, with a highest average range of 54.05%, as illustrated in Table 4. CPU usage for each function is shown in Figure 13. Memory consumption across all tested functions averaged between 672 MB and 775 MB, with the highest memory usage of 1,020 MB observed for the GetVideo function, as shown in Figure 14. Based on traffic data, RegisterVideo registered the highest average traffic at 30.5 MB, whereas CompareVideo registered the lowest average traffic at 28.7 MB. For data traffic, CompareVideo’s minimum was 12 MB and RegisterVideo’s maximum was 20.8 MB. Figure 15 displays the traffic data results. From the analysis results, it is evident that the proposed framework guarantees stable performance in stress testing, which can be employed in scalable, efficient, and reliable video services.

- **Regarding computational and storage overhead:** The pHash value is computationally lightweight and more effective than pixel-wise or feature-based matching. Here, the frame-by-frame comparison involves only bitwise operations to calculate the Hamming distance, which is very effective.
- **Storage Resources:** To store the hash values of frames, we use decentralized storage—namely, the IPFS—to minimize resource overhead. To store videos, we use traditional databases and MySQL. Hashing 1,000 frames requires approximately 8 KB of storage, which is negligible compared to the storage of the original video.

This approach achieves a balanced combination of robustness, resource efficiency, and scalability for video copyright protection.

## 7 Conclusion

The study successfully demonstrates that a fully decentralized, blockchain-based video copyright protection system is both feasible and effective. This can be achieved using blockchain: when video creators securely register on the blockchain and owners verify their ownership of any piece of content through this mechanism, any alteration or piracy can be easily identified. This study validates the performance of a reliable and efficient system, featuring video registration, verification, and comparison modules that run as designed. It provides a transparent and tamper-proof platform for the open and secure management of copyrights, an essential aspect of copyright protection and intellectual property management.

In the future, additional advanced methods of video analysis can be added to make a more accurate comparison of the contents. In addition, incorporating machine learning algorithms could enable more efficient detection of subtle modifications or plagiarized content. Expanding the platform to other forms of digital media will further increase the number of potential use cases for this blockchain platform. Building an easy-to-use front-end interface will greatly extend the target group and, by that means, foster further diffusion. Deploying the system on a public blockchain network will increase the system’s scalability manyfold, with real-world testing across various scenarios. These improvements could solidify the system’s role in protecting intellectual property rights in the evolving digital landscape.

## Data Availability

The original contributions presented in the study are included in the article/supplementary material, further inquiries can be directed to the corresponding author.
